# New Insights into the Antimicrobial and Wound-Healing Properties of Turmeric-Powder-Derived *Curcuma longa* Extracts for Oral-Health-Oriented Applications

**DOI:** 10.3390/biomedicines14051078

**Published:** 2026-05-09

**Authors:** Dana-Emanuela Pitic (Coţ), Andreea Kiş, Ciprian Stroia, Ioana-Cristina Talpoş-Niculescu, Ramona-Amina Popovici, Codruţa-Eliza Ille, Alfred Mark Sallai, Alina Anton, Elena-Alina Moacă, Emilia Daliana Muntean, Maria Suciu

**Affiliations:** 1 Faculty of Dental Medicine, “Victor Babes” University of Medicine and Pharmacy, Revolutiei Ave. 1989, No. 9, 300580 Timisoara, Romaniakis.andreea@umft.ro (A.K.); ille.codruta@umft.ro (C.-E.I.); sallai.mark@umft.ro (A.M.S.); 2 Doctoral School, “Victor Babes” University of Medicine and Pharmacy, Revolutiei Ave. 1989, No. 9, 300580 Timisoara, Romania; 3 Faculty of Agriculture, University of Life Sciences “King Mihai I” from Timisoara, 119 Calea Aradului, 300645 Timisoara, Romania; ciprian.stroia@usvt.ro; 4 Research Center of Digital and Advanced Technique for Endodontic, Restorative and Prosthetic Treatment (TADERP), “Victor Babes” University of Medicine and Pharmacy, Revolutiei Ave. 1989, No. 9, 300580 Timisoara, Romania; 5 Faculty of Pharmacy, “Victor Babeş” University of Medicine and Pharmacy, 2 Eftimie Murgu Square, 300041 Timisoara, Romania; dolghi.alina@umft.ro (A.A.); alina.moaca@umft.ro (E.-A.M.); 6 Research Center for Pharmaco-Toxicological Evaluations, “Victor Babeş” University of Medicine and Pharmacy, 2 Eftimie Murgu Square, 300041 Timisoara, Romania; 7 Faculty of Medicine, “Victor Babes” University of Medicine and Pharmacy, 2 Eftimie Murgu Square, 300041 Timisoara, Romania; emilia.muntean@umft.ro; 8 National Institute for R&D of Isotopic and Molecular Technologies, 67-103 Donat Street, 400293 Cluj-Napoca, Romania; suciu.maria@ubbcluj.ro

**Keywords:** turmeric-derived extracts, antioxidant activity, oral pathogens, oral biofilms, phenolic profile, epithelial compatibility, HaCaT keratinocytes, cytotoxicity

## Abstract

**Background/Objectives**: *Curcuma longa* is widely recognized for its antioxidant, antimicrobial, and wound-related biological effects. The present study aimed to compare two extracts prepared from organic turmeric powder (*Curcuma longa*), using distilled water (CUR-H_2_O) and 96% ethanol (CUR-EtOH), in terms of extraction yield, phytochemical profile, antimicrobial activity, and in vitro biological behavior relevant to future oral-health-oriented applications. **Methods**: The extracts were prepared by maceration followed by ultrasound-assisted processing, concentration, and lyophilization. Their antioxidant potential (AOP) was evaluated by DPPH assay, total phenolic content (TPC) by the Folin–Ciocalteu method, and targeted polyphenolic profile by UHPLC-MS. Antimicrobial activity was assessed by broth microdilution against *Streptococcus mutans*, *Streptococcus oralis*, and *Candida albicans*. In vitro biological activity was investigated on HaCaT keratinocytes. **Results**: CUR-EtOH extract showed a higher extraction yield than CUR-H_2_O (5.13% vs. 2.01%), higher AOP (69.54 ± 0.49% vs. 53.35 ± 0.30%), and a higher TPC (163.87 ± 0.32 vs. 78.05 ± 0.28 mg GAE/g dry extract). Consistent with these TPC results, UHPLC-MS revealed a richer targeted polyphenolic profile in CUR-EtOH extract, particularly in terms of p-coumaric and ferulic acid derivatives. CUR-EtOH extract was more active against the tested oral streptococci, especially *S. mutans* (MIC 10 µL vs. 60 µL for CUR-H_2_O), whereas CUR-H_2_O extract showed a slightly better antifungal effect against *C. albicans* (MIC 60 µL vs. 80 µL). In HaCaT cells, CUR-H_2_O extract exhibited the more favorable compatibility profile, while CUR-EtOH extract showed stronger cytotoxicity, despite promoting faster wound-gap closure at 10 µg/mL. **Conclusions**: The extraction solvent strongly influenced both the chemical profile and biological behavior of the turmeric-powder-derived extracts. These findings suggest that solvent selection may be used to tailor the balance between antimicrobial efficacy and epithelial compatibility in future turmeric-powder-derived preparations intended for oral-health-oriented applications.

## 1. Introduction

The oral cavity is colonized by complex, multispecies, and spatially organized biofilms. Their development follows a reproducible ecological succession, in which early colonizers, particularly streptococci, adhere to host-conditioned surfaces and help establish a structural scaffold for later community members through coaggregation and metabolic/chemical communication [1,2,3,4,5]. In this context, *Streptococcus oralis* is a widespread commensal, frequently described as an initial/pioneer colonizer of dental and implant-associated biofilms, as well as a dominant component of the healthy mucosal microbiota. Moreover, it is involved in surface-colonization processes that may allow subsequent biofilm maturation and the formation of a community associated with oral disease [1,2,6,7,8]. These oral diseases may arise when this homeostatic balance is disrupted, leading to dysbiosis. Dental caries is a biofilm-mediated disease in which *S. mutans* is strongly associated with severe lesions, especially in childhood. Multispecies biofilm studies have shown that the loss or absence of *S. oralis* may permit the overgrowth of *S. mutans*, whereas specific *S. oralis* isolates may antagonize *S. mutans* and reduce enamel demineralization through competitive hydrogen peroxide-related interactions [9,10]. *Candida albicans*, a frequent oral colonizer and opportunistic pathogen, also participates in interkingdom biofilm formation together with streptococci. Interactions between *C. albicans* and *Streptococcus* spp. may enhance mixed-biofilm growth and pathogenic potential, increasing mucosal tissue damage and compromising epithelial integrity [1,11,12,13,14]. Host responses to oral biofilms are likewise multifactorial. In organotypic mucosal and peri-implant epithelial models, commensal *S. oralis* biofilms may trigger host stress-response programs together with relatively attenuated inflammatory mediator production. However, direct exposure of deeper connective tissue cells, such as fibroblasts, to *S. oralis* biofilms may rapidly induce injury, highlighting the importance of epithelial barrier integrity in maintaining host–biofilm homeostasis [7,8,12]. Because biofilm-mediated oral infections are difficult to eradicate, antibiotics alone are often insufficient against biofilm-associated resistance and raise concerns regarding drug resistance and collateral effects on beneficial microbiota. Therefore, there is a clear rationale for novel locally applied interventions that combine antibiofilm and antimicrobial efficacy with host-oriented functions, including anti-inflammatory, barrier-protective, antioxidant, and pro-reparative effects. Such approaches are particularly relevant in the oral mucosal environment, where microbial redox chemistry and tissue stress pathways may contribute to disease progression. These strategies may include natural or bioinspired approaches, including commensal-derived interventions adapted to the oral mucosal environment [7,8,9,15,16].

*Curcuma longa* L. (turmeric) is a rhizomatous species of the Zingiberaceae family, widely recognized both as a culinary plant and as a medicinal resource due to its topical and systemic use, including antiseptic, analgesic, anti-inflammatory, and wound-healing purposes [17,18]. Its medical relevance is closely related to the diverse phytochemical composition of the turmeric rhizome. This includes (i) a characteristic curcuminoid fraction dominated by curcumin (diferuloylmethane), together with demethoxycurcumin and bisdemethoxycurcumin, and (ii) volatile components, such as turmerone, atlantone, and zingiberene, as well as other associated compounds that may contribute to the overall biological profile [19,20,21,22]. Among these constituents, curcumin is identified as the principal phytochemical of turmeric and is associated with many of the pharmacological effects attributed to turmeric-based preparations [19,23,24,25,26]. Curcumin is recognized as a multifunctional natural agent with antioxidant, anti-inflammatory, and antimicrobial properties, including the ability to modulate oxidative stress and inflammatory signaling mediators involved in chronic inflammatory conditions [19,20,21,25,27]. Moreover, turmeric-derived compounds have been associated with biologically relevant actions related to tissue repair, such as processes involved in re-epithelialization, collagen deposition/remodeling, and granulation/angiogenesis, which further supports continued research into their potential in healing-oriented contexts [18,19,22,27]. These actions have generated increasing interest in curcumin as a natural adjunctive or alternative approach in oral medicine and dentistry. Potential applications include biofilm- and inflammation-associated conditions such as dental caries, gingivitis, and periodontitis, as well as inflammatory disorders of the oral mucosa, including oral lichen planus, treatment-related oral mucositis, and oral submucous fibrosis. Delivery strategies have most often focused on mouthwashes, gels, and subgingival or other local applications [19,23,25,26,27,28]. At the same time, current studies in oral and dental health emphasize that, despite the substantial experimental and clinical interest, the evidence base for different indications remains heterogeneous, and further rigorous, long-term clinical research is still required to establish efficacy, optimal formulations, and safety in routine practice [25,27,28,29].

In this context, *Curcuma longa* and its main curcuminoid, curcumin, have attracted considerable interest because of their reported antioxidant, antimicrobial, anti-inflammatory, and wound-related biological effects [30,31,32,33,34,35]. However, these activities are highly dependent on the type of preparation used, since turmeric is not composed of curcumin alone, but of a broader phytochemical matrix whose biological behavior varies according to solvent choice, extraction procedure, and analytical characterization [30,33,36,37,38]. Comparative studies indicate that aqueous, ethanolic, hydroalcoholic, methanolic, and formulated preparations may differ substantially in their antioxidant performance and broader pharmacological activity, most likely because they recover different groups of bioactive constituents [30,37,38,39]. In this context, the extraction solvent is not merely a technical parameter, but an important determinant of the final chemical profile and, consequently, of the measurable biological effects [30,36,37,38,39]. At the biological level, the literature supports a promising but heterogeneous profile for turmeric- and curcumin-based preparations. Antioxidant activity is frequently reported, but its magnitude, and in some cases even its direction, may vary with assay conditions, dose, formulation, and tissue context [30,34,36,37,38,39,40]. Likewise, antimicrobial effects are widely described for turmeric extracts and curcumin, although these outcomes appear to be strongly influenced by preparation type, matrix, and delivery format [32,34,35,37]. A similar context dependence has also been described for wound-related effects: while curcumin-containing topical systems have been associated with improved skin repair and re-epithelialization, aqueous and alcoholic preparations may behave differently depending on the tissue model and biological endpoint evaluated [31,33,36,40]. Taken together, these observations suggest that the reported biological activities of *C. longa* preparations should be interpreted in relation to how the extract was obtained and in which experimental model it was tested, thereby justifying comparative studies focused on solvent-dependent differences.

Comparing aqueous and ethanolic extracts is scientifically justified because solvent choice is one of the main factors governing extraction yield and chemical composition. Since phytochemicals differ widely in polarity and solubility, water and ethanol recover different fractions from the same biological matrix, often resulting in chemically distinct extracts rather than simple quantitative differences in extracted mass. Reviews on plant extraction have emphasized that solvent selection is a major determinant of extract composition, stability, and downstream biological properties [41,42]. This principle is particularly relevant for turmeric- and polyphenol-rich preparations. Comparative studies have shown that ethanol-containing systems frequently improve the recovery of phenolic compounds and antioxidant-associated constituents compared with water alone, although the exact outcome remains matrix-dependent. In turmeric, different extraction solvents have been associated with marked differences in total phenolic content and antioxidant activity, while studies on other natural matrices likewise confirm that solvent composition can significantly alter polyphenolic profiles and measurable bioactivity. Therefore, the comparison between aqueous and ethanolic *Curcuma longa*-derived extracts is scientifically relevant because it helps explain how solvent-dependent chemical differences may translate into different antioxidant and biological effects [43,44].

Nevertheless, although turmeric- and curcumin-based preparations have been extensively investigated, the available literature still shows several important gaps. Most studies have focused on isolated aspects, such as antioxidant activity, antimicrobial effects, or wound-related properties. Fewer investigations have integrated extraction yield, phytochemical profile, antioxidant potential, antimicrobial activity, and epithelial biological response within the same comparative design. In addition, many published studies were performed on turmeric rhizome powder or purified curcumin, while fewer data are available for turmeric-powder-derived extracts obtained with different solvents and evaluated in relation to oral-health-oriented applications. The present study was designed as a comparative preclinical model intended to clarify whether solvent-dependent extraction of a commercial turmeric powder starting material translates into meaningful differences in antimicrobial efficacy, antioxidant-related profile, and epithelial compatibility relevant to oral-health-oriented use. Accordingly, the relationship between solvent-dependent composition, antimicrobial performance, and epithelial compatibility still requires further clarification.

In this context, the present study aimed to comparatively evaluate two turmeric-powder-derived extracts prepared in distilled water (CUR-H_2_O) and 96% ethanol (CUR-EtOH). The comparison included extraction yield, antioxidant potential, total phenolic content, targeted polyphenolic profile, antimicrobial activity against selected oral and opportunistic microorganisms, and in vitro biological behavior on HaCaT keratinocytes. More specifically, the study sought to determine how solvent-dependent extraction influences the chemical composition and biological performance of the resulting preparations, in order to explore their potential for future oral-health-oriented applications. The present comparative design was intentionally restricted to two contrasting solvent systems, namely distilled water and 96% ethanol, in order to provide an initial polarity-based comparison rather than a full solvent-optimization study.

## 2. Materials and Methods

### 2.1. Turmeric-Powder-Derived Extract Preparation and Extraction Yield Assessment

A commercially available organic turmeric powder (*Curcuma longa*) was acquired from a local supplier (S.C. Republica Bio-Curcuma S.R.L., Lot P242149, Timisoara, Romania) and stored at 22 ± 2 °C until processing. According to the product label, the material consisted of 100% Curcuma powder (*Curcuma longa*), with no declared curcuminoid standardization. Distilled water and 96% ethanol (*v*/*v*), purchased from Chemical Company SA (Iasi, Romania), were used to obtain the turmeric-powder-derived extracts. The extraction methodology was performed according to the protocol reported by Shakeri et al. [45] and Abdel-Shafy et al. [46], with slight modifications. Briefly, 12.5 g of organic turmeric powder was mixed with the solvent (distilled water and 96% ethanol) to maintain a mass-to-volume ratio of 1:10. Both mixtures were left to macerate for 7 days at room temperature (22 ± 2 °C). The 7-day maceration period was selected a priori as a mild room-temperature extraction step adapted from the published protocols, with the intention of allowing sufficient solvent–matrix contact before ultrasound-assisted processing. This extraction time was applied equally to both solvent systems in order to maintain a controlled comparative design. After the 7-day maceration period, both extracts were subjected to ultrasound-assisted processing, but under different conditions. The ethanolic extract was probe-sonicated for 30 min (50% amplitude, 10 s pulse/on and 5 s pulse/off) using a QSonica Q700 sonicator (Qsonica L.L.C., Newtown, CT, USA), then filtered using a Rocker 400 Oil Free Vacuum Pump (Rocker Scientific Co., Ltd., Taipei, Taiwan) and concentrated under reduced pressure at 40 °C using a rotary evaporator (Heidolph G3, Schwabach, Germany). In parallel, the aqueous extract was sonicated for 60 min in an Elma S120 Elmasonic ultrasonic water bath (Elma Ultrasonic BV, Wingene, Belgium) at 40 °C, and was subsequently concentrated under the same conditions as the ethanolic extract. Because probe sonication and ultrasonic water-bath treatment differ in the mode and intensity of energy transfer, the 30 min and 60 min processing times should not be interpreted as directly equivalent ultrasound doses. These post-maceration conditions were applied as part of the solvent-specific extraction workflow used in the present study, rather than as a fully parameter-matched ultrasound optimization design. Until further use, each crude extract was subsequently lyophilized and stored at 4 °C in a glass container.

Both lyophilized extracts prepared from commercially available turmeric powder derived from *Curcuma longa* (further denoted as CUR-H_2_O and CUR-EtOH) were kept in a refrigerator at 4 °C until further use (characterization and biological assessment). The extraction yield was assessed for each extract, taking into account the extraction time, turmeric powder/solvent ratio, contact time between turmeric powder and solvent, as well as solvent concentration. To calculate the extraction yield, a precise volume of each extract obtained (aqueous and ethanol) was subjected to an evaporator at a constant temperature of up to 40 °C to avoid phytocompound degradation. Using the following equation (Equation (1)), the extraction yield was calculated:

(1) $$\eta \;\left[\%\right]=\;\left(\frac{m_{solid\;residue}\;\times \;V_{extract}}{V_{total}\;\times \;m_{curcumin\;powder}}\right)\;\times 100$$

where *η* [%] represents the extraction yield [%]; $m_{solid\;residue}$ is the mass of the solid residue obtained after extract concentration (g); $V_{extract}$ is the volume of each extract sample subjected to concentration process (mL); $V_{total}$ is the total volume of each extract sample obtained after the extraction process (mL); and $m_{curcumin\;powder}$ is the amount of the turmeric powder taken into account (g).

Extraction efficiency was assessed only at the final endpoint and was not monitored at intermediate time points; therefore, the present protocol should be interpreted as a comparative extraction workflow rather than a time-optimization study.

Before testing, the lyophilized extracts were reconstituted in distilled water and ethanol to obtain the desired stock concentrations.

### 2.2. Nutraceutical Characterization of the Extracts

#### 2.2.1. Antioxidant Potential (AOP)

Free radical scavenging activity was assessed using the DPPH assay, employing a freshly prepared 0.3 mM DPPH (1,1-diphenyl-2-picrylhydrazyl) solution in 70% ethyl alcohol (Sigma-Aldrich Chemie GmbH, Munich, Germany), according to the previously reported protocol [47]. This technique is frequently used to assess the antioxidant potential of various extracts, due to their ability to donate electrons or hydrogen atoms to free radicals. The DPPH radical, which is deep purple in color due to its unpaired electron, loses this color when reduced to the hydrazine form, turning pale yellow. To determine the antioxidant capacity, 1 mL of each extract was mixed with 2.5 mL of 0.3 mM DPPH solution. The mixture was kept in the dark at room temperature for 30 min. After this time interval, the absorbance of each sample was measured at a wavelength of 517 nm using a UV–Vis spectrophotometer (Specord 205; Analytik Jena AG, Jena, Germany). Each extract was analyzed in triplicate as technical analytical replicates, and the results were presented as the mean ± standard deviation. Because the aim of the present DPPH assay was to provide a standardized comparative screening between the two crude extracts, antioxidant activity was evaluated at a single working concentration derived from the reconstituted stock solutions, rather than through a serial dilution design for IC_50_ determination. As a control, a sample was used in which the extract volume was replaced with 70% ethyl alcohol. The free radical scavenging capacity was expressed as a percentage using the formula:

(2) $$AOP\;\left[\%\right]=(\frac{{Abs}_{control}-\;{Abs}_{sample}}{{Abs}_{control}})\times 100$$

where $AOP\;\left[\%\right]$ represents the antioxidant activity measured for each extract [%]; ${Abs}_{control}$ represents the absorbance value of the DPPH solution without the extract; and ${Abs}_{sample}$ corresponds to the absorbance of the sample analyzed in the presence of the DPPH free radical.

#### 2.2.2. Total Phenolic Content (TPC)

The total polyphenol content was determined using a slightly modified Folin–Ciocalteu method [48]. The extracts obtained were diluted at a ratio of 1:100, and 0.5 mL of this dilution was mixed with 1.25 mL of Folin–Ciocalteu reagent (Sigma-Aldrich, Munich, Germany), which had been previously diluted 1:10 with distilled water. After a 5 min reaction period at room temperature, 1 mL of 7.5% Na_2_CO_3_ solution (Geyer GmbH, Renningen, Germany) was added. The resulting mixture was incubated for 30 min at 50 °C in a thermostat (INB500, Memmert GmbH, Schwabach, Germany). Subsequently, the absorbance of each extract was measured at a wavelength of 760 nm using a Specord 205 spectrophotometer (Analytik Jena Inc., Jena, Germany). Each extract was analyzed in triplicate as technical analytical replicates, and the results were presented as mean ± standard deviation. The quantification of the TPC was done according to the equation resulting from the calibration curve (R^2^ = 0.998), performed using gallic acid standard solutions (Fluka, Madrid, Spain) at different concentrations ranging from 0 to 200 µg/mL. The results were expressed as milligrams of gallic acid equivalents (GAEs) per gram of dry extract. Because the Folin–Ciocalteu reagent responds to reducing substances in addition to phenolic compounds, the obtained values should be interpreted operationally as Folin–Ciocalteu-reactive reducing capacity expressed as gallic acid equivalents, rather than as an absolute quantification of phenolics alone.

#### 2.2.3. Ultra-High-Performance Liquid Chromatographic (UHPLC) Analysis

For the UHPLC analysis, ultrapure water, methanol, and formic acid were purchased from VWR International Europe Services S.R.L. (Bucharest, Romania). Native reference compounds used for calibration were acquired from Dr. Ehrenstorfer GmbH (Augsburg, Germany) and Toronto Research Chemicals Inc. (Vaughan, ON, Canada), while the corresponding labeled standards were purchased from the same manufacturers. The analytical standards used in the present study were: trans-p-Coumaric acid (DRE-C11734100), trans-Ferulic acid (DRE-C13644100), (E/Z)-Ferulic acid (DRE-C13644090), 3,4-Dihydroxybenzoic acid (DRE-C12634710), Rutin (DRE-C16880000), Quercetin (DRE-C16695000), Caffeic acid (DRE-C10934700), Chlorogenic acid (DRE-C11415750), Rosmarinic acid (DRE-C16819500), Gallic acid (DRE-C13998280), and Genistein (DRE-C13999800) and from Toronto Research Chemicals Inc. (Ontario, Canada): p-Coumaric acid (C755365), 3,5-Dihydroxybenzoic Acid (D451700), Resveratrol (R150000), and 2,4-Dihydroxybenzoic Acid (D678940). Labeled standards were purchased from the same manufactures as follows: Toronto Research Chemicals—trans-p-Coumaric-d6 Acid (C755392), Ferulic Acid-d3 (F308902), 3,4-Dihydroxybenzoic Acid-d3 (TRC-D451682), Rutin-d3 (R701800), Quercetin-d3 (Q509502), Caffeic Acid-13C3 (C080002), Chlorogenic acid 13C3 (TRC-C366542), Genistein d4 (G350010), and Resveratrol 13C6 (R150002) and Gallic acid D2 (DRE-C13998281) from Dr. Ehrenstorfer.

In order to identify and quantify each polyphenol, an ultra-high-performance liquid chromatographic (UHPLC) system from Shimadzu connected to an LCMS 8045 triple quadrupole mass spectrometer was used, according to a previously validated and described method [49]. The configurations of the equipment include a solvent delivery pump, a degassing unit, a column oven, an autosampler, a system controller, and a triple quadrupole mass spectrometer for identification. The LabSolutions Ver. 5.86 software was used to analyze results. For this purpose, the aqueous and alcoholic extracts were centrifuged at 18,000 rpm, and 980 µL of the supernatant with 20 µL of internal standard (at a concentration of 25 µg/mL) was transferred into an HPLC vial, and 5 µL of each mix was injected. The polyphenols were separated using a C18 reversed-phase column. The mobile phase A contains ultrapure water with 0.05% formic acid solution (*v*/*v*). Mobile phase B consists of methanol with 0.05% formic acid solution (*v*/*v*). Formic acid was used as an organic modifier. The gradient starts predominantly with the aqueous phase and 10% methanol up to 5 min, continues with the organic phase, 95% methanol up to 8 min, and finishes with 10% methanol up to 14 min, with a flow rate of 0.4 mL/min at 40 °C. MS detection uses an electrospray ionization (ESI) source.

The chromatographic method has been developed for the determination of 15 polyphenols and provides a simultaneous analysis of these by a single-pass column. The compounds detected by the MS in the samples were quantified using the calibration curve ranging from 50 µg/mL to 5000 ng/mL, with good linearity (R^2^ > 0.998). The limit of quantification was 50 ng/mL, and the limit of detection was 15 ng/mL. The applied UHPLC-MS method was intentionally restricted to a targeted panel of 15 low-molecular-weight polyphenols. Curcuminoids such as curcumin, demethoxycurcumin, and bisdemethoxycurcumin were not included in this analytical panel and, therefore, were not quantified by this assay. The validation parameters obtained for the applied UHPLC-MS method showed a mean recovery of 101.03 ± 6.45%, while method precision, expressed as relative standard deviation (RSD), ranged from 0.88% to 14.53%, with a mean RSD of 5.08%. These values fall within commonly accepted analytical validation criteria and support adequate accuracy and precision for the quantification of the investigated compounds under the applied conditions. UHPLC-MS quantification was performed using triplicate analytical measurements of the same extract preparation.

### 2.3. Antimicrobial Activity

The antimicrobial activity of the extracts prepared from commercially available turmeric powder derived from *Curcuma longa* was evaluated by the broth microdilution method through determination of the minimum inhibitory concentration (MIC) and measurement of microbial growth by optical density (OD), in accordance with internationally accepted susceptibility testing principles and broth dilution procedures [50,51]. Optical density measurements were performed according to ISO 20776-1:2019 [52]. The MIC was defined as the lowest tested volume of extract that produced visible inhibition of microbial growth, and was supported by the corresponding reduction in optical density relative to the untreated growth control. The antimicrobial assay was performed against *Streptococcus mutans* ATCC 25175, *Streptococcus oralis* ATCC 9811, and *Candida albicans* ATCC 10231. All culture media and analytical-grade reagents used in the microbiological analyses were purchased from Sigma-Aldrich Chemie GmbH (Munich, Germany) and Geyer GmbH (Renningen, Germany). The tested microbial strains were obtained from the American Type Culture Collection (ATCC) (Manassas, VA, USA).

The aqueous and ethanolic turmeric-powder-derived extracts were tested using the broth dilution protocol previously described in related studies [53,54,55,56]. *S. mutans* and *S. oralis* were cultured on Mitis Salivarius Agar (Oxoid Limited, Thermo Fisher Scientific Inc., Waltham, MA, USA) supplemented with 1% potassium tellurite and incubated under anaerobic conditions at 37 °C for 48 h. *C. albicans* was cultured on Sabouraud Dextrose Agar (SDA) and incubated under aerobic conditions at 37 °C for 24–48 h. After incubation, fresh colonies were used to prepare the microbial inocula. The inocula were adjusted to a turbidity equivalent to the 0.5 McFarland standard (approximately 1.5 × 10^8^ CFU/mL). Working microbial suspensions were then prepared in sterile Brain Heart Infusion (BHI) broth by diluting the fresh cultures 10^−2^ (1:100) for the *Streptococcus* strains and 10^−3^ (1:1000) for *C. albicans*. Subsequently, 100 µL of each microbial suspension was transferred into the wells of sterile 96-well microdilution plates using a digital multichannel pipette.

Different volumes of each extract stock solution were added to the inoculated wells for MIC screening (10, 20, 40, 60, 80, and 100 µL per well). Because the assay was designed using defined stock-extract volumes, MIC values were initially expressed as the lowest tested stock-extract volume per well associated with growth inhibition. To improve comparability, the corresponding mass-equivalent doses were also calculated. Based on the stock concentrations used for reconstitution, namely 97.8 mg/mL for CUR-EtOH and 98.2 mg/mL for CUR-H_2_O, the tested volumes corresponded to 0.978, 1.956, 3.912, 5.868, 7.824, and 9.780 mg/well for CUR-EtOH and 0.982, 1.964, 3.928, 5.892, 7.856, and 9.820 mg/well for CUR-H_2_O, respectively. Untreated microbial suspensions in BHI broth served as the positive growth control, while sterile BHI broth served as the blank control. Ampicillin was included as a bacterial reference/control condition for the antibacterial part of the assay. However, this control was not used as a harmonized positive efficacy comparator across the full microbial panel, because no matched antifungal reference agent (such as nystatin or fluconazole) was included for *Candida albicans* within the same experimental framework. Therefore, the assay was interpreted primarily as a comparative screening between the two crude extracts under identical test conditions. In addition, to correct for the intrinsic absorbance and color of the extracts, extract-containing wells prepared under the same conditions but without microbial inoculum were included, and their absorbance values were subtracted from the corresponding sample readings. Thus, the antimicrobial activity was interpreted on the basis of corrected OD values (OD_sample − OD_extract background). This correction was particularly important because the tested extracts were colored and could otherwise interfere with the spectrophotometric reading.

After preparation, the microdilution plates were incubated for 24 h at 37 °C under aerobic conditions. Microbial growth was quantified by measuring the optical density at 540 nm (OD540) using a Bio-Rad PR1100 microplate spectrophotometer (Bio-Rad, Hercules, CA, USA). Before each measurement, the instrument underwent routine calibration and blank correction using sterile BHI medium. All samples were analyzed in triplicate as technical replicate wells within the same experimental setup, and the results were expressed as mean OD values ± standard deviation.

The microbial growth rate (bacterial/mycelial growth rate (BGR/MGR)) was calculated relative to the positive growth control according to the following equation:

(3) $$Growth\;rate\;(BGR/MGR)\;\left[\%\right]=\;\frac{{OD}_{sample}}{{OD}_{positive\;growth\;control}}\;\times \;100$$

The microbial inhibition rate (bacterial/mycelial inhibition rate (BIR/MIR)) was calculated as:

(4) $$Inhibition\;rate\;\left(BIR/MIR\right)\left[\%\right]=100\;-Growth\;rate\;(BGR/MGR)$$

where ${OD}_{sample\;corrected}$ represents the optical density of the inoculated well after subtraction of the extract background, and ${OD}_{positive\;growth\;control}$ represents the optical density of the untreated inoculated control.

The MIC was recorded as the lowest tested stock-extract volume associated with inhibition of microbial growth under the experimental conditions used [57]. For easier interpretation, the corresponding crude-extract mass per well was also considered in the presentation of the results.

### 2.4. In Vitro Assessments

#### 2.4.1. Cell Line and Cell Culture Media

Immortalized human keratinocyte cell line (HaCaT, CLS 300493) was purchased from CLS—Cell Line Services (Eppelheim, Germany), and was grown in DMEM media (high glucose Dulbecco’s Modified Eagle’s Medium), containing 4.5 g/L glucose, 4 mM L-Glutamine, 3.7 g/L NaHCO_3_, 1.0 mM sodium pyruvate, and supplemented with 10% fetal calf serum (Biowest) and 1% Penicillin–Streptomycin. Cells were kept at 37 °C, 5% CO_2,_ and ≥95% humidity. When reaching the exponential phase, cells were detached with trypsin, counted using a hemacytometer, and plated in 96-well plates at a seeding density of 10^4^ cells/well. After 24 h, compounds to be tested were added to the wells at final concentrations of 10, 25, 50, 75, and 100 µg/mL. Controls included untreated cells, 10% Tween 20, 1% ethanol, and 10% ethanol. Each concentration was tested in eight replicate wells within the same assay. HaCaT cells were selected as a robust and reproducible immortalized epithelial model suitable for comparative in vitro screening, while acknowledging that they do not fully replicate oral mucosal keratinocytes.

#### 2.4.2. MTT Protocol: Mitochondrial Activity Assessment

The MTT reagent (3-[4,5-dimethylthiazol-2-yl]-2,5-diphenyl tetrazolium bromide) was employed to assess the cellular viability, quantified via colorimetric assay. The MTT cellular viability test measures cellular viability through mitochondria metabolism [58]. In brief, cells were left in contact with the test samples for 24 and 48 h, and then 0.5 mg/mL MTT solution was added to each well, and incubated for 1.5 h at 37 °C. Afterwards, the cells were solubilized with propanol, and the plate was read in the ELISA plate reader (BioTek Instruments Inc., Winooski, VT, USA) at 550 and 630 nm. Mitochondrial activity of the cell population was reported to the untreated control, which was considered to be 100% at the analyzed time points. Statistical intergroup differences were calculated with ANOVA single factor followed by Tukey’s test, and values of *p* < 0.05 were considered relevant.

#### 2.4.3. LDH Release Method: Cytotoxicity Test

For quantifying the released lactate dehydrogenase (LDH) enzyme, 50 µL of culture medium was transferred from each well of the plates with MTT, before analysis, to new 96-well plates [59]. To these samples were added 50 µL each of solutions of lithium lactate, nicotine adenine dinucleotide, and phosphate-buffered saline, to a final volume of 200 µL per well. Each concentration was tested in eight replicate wells within the same assay. After 5 min of incubation, the plates were read at 490 and 630 nm in the spectrophotometer. The LDH concentration was calculated in enzymatic units per volume and time, using the following formula:

(5) $$[mU/mL/min]\;=\;\left(\frac{∆A/\min\;\times \;V_{t}}{\varepsilon \times d\times V_{p}}\right)\;\times \;1000$$

where $∆A/\min\;=\;\frac{{Abs}_{min\;5}}{5\;min}$; $V_{t}$ is the reactive volume per well (200 µL); ε is the extinction coefficient of iodonitrotetrazolium (18.4 mM^−1^cm^−1^); and d is the light path (6.4 mm). Statistical intergroup differences were calculated with ANOVA single factor followed by Tukey’s test, and values of *p* < 0.05 were considered relevant.

#### 2.4.4. Wound Healing Assay

Cells were seeded in 12-well plates at a density of 10^5^ cells/well and were left to reach 100% confluency for 48 h. Using a 1 mL blue pipette tip (tip diameter 1.5 mm), a scratch was made across the surface of the monolayer. The medium was then discarded, and 0.5 mL fresh medium with the 10 µg/mL compounds was added to each well. Controls included untreated cells, 10% Tween 20, 1% ethanol, and 10% ethanol. Each test condition was made in duplicate. Images were taken from approximately the same region using an AmScope Inverted trinocular microscope with a camera attached, at 4× magnification, at 24, 48, and 72 h time points, until wound closure was observed. At each time point, the medium was discarded, and 0.5 mL of fresh medium with the 10 µg/mL compounds was added to avoid false results from spent medium, due to high confluency. Wound opening was measured using ImageJ software (version number 1.53f, https://imagej.nih.gov/ij/, accessed on 14 March 2026) for least 4 points per image, and the cell’s ability to migrate and repopulate the gap was calculated in Excel. Statistical intergroup differences were calculated with ANOVA single factor followed by Tukey’s test, where values of *p* < 0.05 were considered relevant. Because this assay was conceived as an exploratory migration screen, duplicate wells were used for each condition, while multiple measurements per image were performed to improve local measurement consistency. Nevertheless, these repeated measurements do not replace independent biological replicates.

### 2.5. Statistical Analysis

All quantitative data were expressed as mean ± standard deviation (SD). For the nutraceutical characterization assays, comparisons between CUR-H_2_O and CUR-EtOH were evaluated using an unpaired Student’s *t*-test in Origin Lab—Data Analysis and Graphing Software version 9.8.5.212, Szeged, Hungary. For the antimicrobial assay, comparisons between the two extracts at the same tested volume and for the same microbial strain were evaluated using an unpaired Student’s *t*-test, where applicable. For the cell-based assays (MTT, LDH, and wound-healing assay), statistical intergroup differences were calculated by one-way ANOVA followed by Tukey’s post hoc test, with *p* < 0.05 considered statistically significant. The statistical analysis was performed using GraphPad Prism software (version 10.2.3; GraphPad Software, San Diego, CA, USA).

## 3. Results

### 3.1. Extraction Yield Assessment

By applying Equation (1), the extraction yield was calculated for 50 mL of each extract. Regarding the aqueous extract (CUR-H_2_O), the extraction yield was 2.01%/50 mL extract, and for the ethanolic one (CUR-EtOH), it was 5.13%/50 mL extract. These results are notable because curcumin is relatively insoluble in water and dissolves much more readily in organic solvents such as ethanol, which means that aqueous extracts recover mainly more polar fractions, while ethanol extracts recover curcuminoids more efficiently. In addition, the protocol employed and experimental conditions for the extraction process play an important role. Under the present conditions, ethanol was the more efficient solvent than water for recovering crude extract from turmeric powder, while the relatively low aqueous yield most likely reflects the poor water solubility of curcumin and the lower efficiency of water, compared with ethanol, in recovering less polar turmeric-associated constituents under the applied conditions.

### 3.2. Nutraceutical Screening

#### 3.2.1. Antioxidant Potential (AOP)

For the antioxidant potential, two stock solutions of each crude lyophilized extract were prepared. The concentration of the CUR-H_2_O stock solution was 0.0982 g/mL, and 0.0978 g/mL was the concentration of the CUR-EtOH stock solution. After applying Equation (2), the results showed that the CUR-H_2_O extract had an AOP of 53.35 ± 0.30%, whereas the CUR-EtOH extract reached 69.54 ± 0.49% AOP. Both extracts showed substantial antioxidant potential, with CUR-EtOH exhibiting higher DPPH scavenging activity than CUR-H_2_O at the tested stock concentrations. However, because the extracts were assayed at almost identical stock concentrations, this difference should be interpreted as an intrinsic potency ranking rather than a concentration-dependent comparison under the present assay conditions. Accordingly, these data represent a single-concentration comparative estimate of antioxidant activity and do not allow direct calculation of IC_50_ values.

#### 3.2.2. Total Phenolic Content (TPC)

For the TPC determination, the same concentration of the extract stock solution was analyzed. In the case of the CUR-H_2_O extract, a TPC of 78.05 ± 0.28 mg GAE/g dry extract was obtained, whereas in the case of the CUR-EtOH extract, a TPC of 163.87 ± 0.32 mg GAE/g dry extract resulted. The TPC of CUR-EtOH extract was approximately 2.1-fold higher than that of CUR-H_2_O extract.

#### 3.2.3. Polyphenols by UHPLC Analysis

The results regarding the polyphenolic compounds present in CUR-H_2_O and CUR-EtOH extracts are exhibited in Table 1. Table 1 reflects only the targeted polyphenols included in the validated UHPLC-MS panel and should not be interpreted as a complete phytochemical composition of the extracts. Curcuminoids were not part of this analytical method and are therefore not reported here.

**Figure 1:**
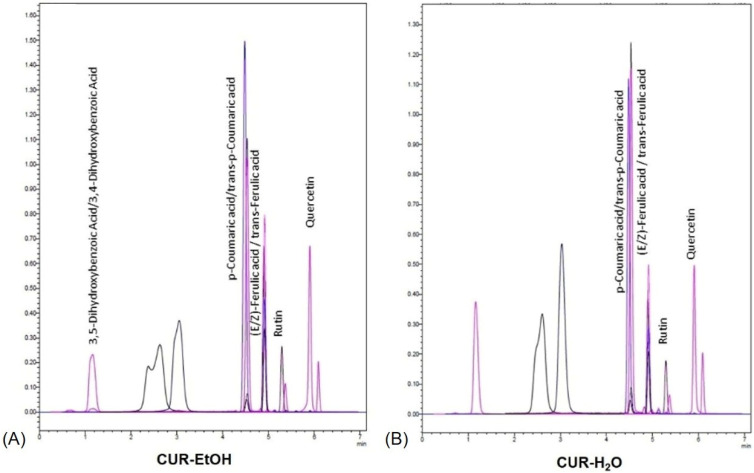
The total ion chromatographic profiles of both extracts. (**A**) CUR-EtOH—ethanolic extract prepared from commercially available turmeric powder (*Curcuma longa*); (**B**) CUR-H_2_O—aqueous extract prepared from commercially available turmeric powder (*Curcuma longa*).

One can observe that polyphenols were extracted in greater quantity in the CUR-EtOH extract, the most abundant being the class of isomers of p-Coumaric/trans-p-Coumaric acid, with a concentration of 5.24 µg/mL. In the CUR-H_2_O extract, the concentration was six times smaller, with the highest concentration of all polyphenols identified in the extract. (E/Z)-Ferulic acid/trans-Ferulic acid were identified in both samples, with a concentration 10 times higher in the alcoholic extract. 3,5-Dihydroxybenzoic Acid/3,4-Dihydroxybenzoic Acid were found only in the CUR-EtOH extract, and rutin and quercetin were identified, with well-defined peaks but still with concentrations below the limit of quantification. Figure 1 shows the total ion chromatograms of the two extracts.

Therefore, the comparative phytochemical interpretation was restricted to the targeted analytes that were reliably detected within the validated method range, while compounds reported as NF or <LOQ were not used as a basis for quantitative comparison.

### 3.3. Antimicrobial Activity

The antimicrobial activity of the two turmeric-powder-derived extracts was evaluated against *Streptococcus mutans* ATCC 25175, *Streptococcus oralis* ATCC 9811, and *Candida albicans* ATCC 10231 by broth microdilution, based on optical density measurements and MIC determination.

#### 3.3.1. Optical Density and Inhibitory Effect

The raw OD values measured after incubation showed a concentration-dependent reduction in microbial growth for both extracts, although the magnitude of the effect varied according to the microbial strain and extraction solvent. In general, CUR-EtOH extract exhibited stronger antibacterial activity than CUR-H_2_O extract against the two oral streptococcal strains, whereas the aqueous extract showed a slightly better effect against *C. albicans* at several tested volumes. The complete raw OD values are presented in Table 2.

After correction for the intrinsic absorbance of the extracts, the same trend remained evident. For *S. mutans*, the corrected OD values for CUR-EtOH decreased from 0.351 at 10 µL to 0.205–0.210 at 80–100 µL, whereas CUR-H_2_O extract decreased from 0.830 at 10 µL to 0.293 at 100 µL, indicating a markedly stronger inhibitory effect of the ethanolic extract. A similar pattern was observed for *S. oralis*, where CUR-EtOH reduced the corrected OD from 1.048 at 10 µL to 0.285 at 100 µL, while CUR-H_2_O extract decreased from 0.820 to 0.366 across the same range. In contrast, for *C. albicans*, CUR-H_2_O extract generally produced lower corrected OD values than CUR-EtOH, particularly at higher tested volumes; for example, at 100 µL, the corrected OD was 0.276 for CUR-H_2_O compared with 0.310 for CUR-EtOH. These corrected OD data are shown in Table 3.

When the inhibitory effect was expressed relative to the positive growth control, CUR-EtOH showed the highest activity against *S. mutans*, with inhibition increasing from 5.9% at 10 µL to 43.8% at 100 µL. Against *S. oralis*, CUR-EtOH also showed a progressive inhibitory effect, although less pronounced, reaching 16.7% at 100 µL. CUR-H_2_O displayed weaker activity against both streptococcal strains, with inhibition values of 21.4% against *S. mutans* and zero or very low inhibition values against *S. oralis* at several tested volumes, indicating limited antibacterial efficacy under the applied conditions. At those tested volumes where the corrected OD exceeded the positive growth control, inhibition was considered absent and is presented as 0.0% in Table 4. For *C. albicans*, both extracts showed moderate antifungal activity, but CUR-H_2_O was slightly more effective than CUR-EtOH at higher tested volumes, reaching 27.6% inhibition at 100 µL compared with 18.6% for CUR-EtOH. These data are summarized in Table 4.

Overall, the results indicate a strain-dependent antimicrobial effect. The CUR-EtOH extract was more active against the two tested bacterial strains, especially *S. mutans*, whereas the CUR-H_2_O extract showed a somewhat better inhibitory effect against *C. albicans*.

#### 3.3.2. Minimum Inhibitory Concentration (MIC)

The MIC values confirmed the stronger antibacterial activity of CUR-EtOH extract and the slightly better antifungal activity of CUR-H_2_O extract. For *S. mutans*, the MIC of CUR-EtOH extract was 10 µL/well, corresponding to approximately 0.978 mg crude extract/well, whereas the MIC of CUR-H_2_O extract was 60 µL/well, corresponding to approximately 5.892 mg/well, indicating substantially greater susceptibility of this strain to the ethanolic extract. For *S. oralis*, CUR-EtOH extract showed a MIC of 100 µL/well (approximately 9.780 mg/well), while no MIC could be established for CUR-H_2_O extract within the tested range. For *C. albicans*, the MIC values were 80 µL/well (approximately 7.824 mg/well) for CUR-EtOH extract and 60 µL/well (approximately 5.892 mg/well) for CUR-H_2_O extract. These data demonstrate that *S. mutans* was the most susceptible microorganism overall, particularly to CUR-EtOH extract, while *S. oralis* was the least susceptible, especially to CUR-H_2_O extract. The MIC values are included in Table 4.

### 3.4. In Vitro Assessment

To better understand the biological behavior of the two extracts in an immortalized epithelial keratinocyte model, the in vitro assessment on HaCaT cells (immortalized human keratinocyte) included three complementary assays: the MTT assay for mitochondrial metabolic activity, the LDH assay for membrane damage/cytotoxicity, and the wound-healing assay for cell migration and repopulation capacity. Overall, the results showed that CUR-H_2_O extract was well tolerated by HaCaT cells over the tested concentration range, whereas CUR-EtOH extract markedly reduced cell viability and increased cytotoxicity. At the lowest tested concentration (10 µg/mL), both extracts could still be further evaluated in the wound-healing model, where they promoted gap closure compared with the untreated control.

#### 3.4.1. Viability Assay

**Figure 2:**
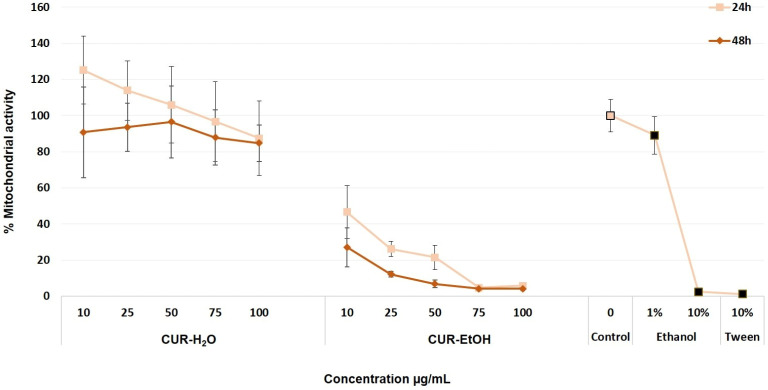
Mitochondrial activity of HaCaT cells treated with CUR-H_2_O and CUR-EtOH extracts. Values are expressed as mean ± SD. Statistical significance was evaluated by one-way ANOVA followed by Tukey’s post hoc test.

The MTT assay was used as a first indicator of how well HaCaT cells tolerated the two extracts, since higher mitochondrial activity reflects better metabolic status and, indirectly, a higher proportion of viable cells. Among the two tested extracts, CUR-H_2_O extract showed the most favorable biocompatibility profile, maintaining HaCaT mitochondrial activity close to or above the untreated control after both 24 and 48 h of exposure (Figure 2). After 24 h, CUR-H_2_O extract at the lowest concentrations tested (10, 25, and 50 µg/mL) determined a mitochondrial activity increase (up to 125.2 ± 18.7%), when compared to untreated control, and the highest ones tested (75 and 100 µg/mL) reduced cell viability down to 87.4 ± 20.5%. At 48 h of exposure, cell mitochondrial activity ranged from 84.5 to 94.6% at the tested concentrations, still indicating biocompatibility. No statistically significant differences were observed for the group of samples treated with CUR-H_2_O extract for 24 h, compared to the untreated control, and for the 25 and 50 µg/mL CUR-H_2_O extract for 48 h compared to the untreated control. These findings indicate that the aqueous extract did not impair the metabolic activity of HaCaT cells in a biologically relevant manner and may even have supported cell metabolic activity at the lower tested concentrations.

In contrast, the ethanolic extract showed a pronounced cytotoxic effect, as reflected by the sharp decrease in mitochondrial activity even at the lowest tested concentration. When cells were treated with the CUR-EtOH extract, their mitochondrial activity dropped significantly to 46.5 (±14.6%) at 10 µg/mL, and then to 5.8 (±1.8%) for 100 µg/mL. After 48 h of exposure, these values were even lower (27.1 at 10 µg/mL down to 4.3%). Because the CUR-EtOH extract was obtained using ethanol, solvent controls were included to exclude the possibility that the observed effects were caused only by the residual solvent. At 1% ethanol, cells presented a slightly reduced mitochondrial activity (89.1 ± 10.4%) and cell population death at 10% ethanol (2.4 ± 0.6%), which indicates that the residual ethanol alone is unlikely to fully explain the effect observed for CUR-EtOH extract (*p* < 0.05) and that cells are indeed sensitive to higher alcohol concentrations. Another cell death control we used was Tween 20 at 10% concentration, which induced population death (1.2 ± 0.1%). Overall, the 1% ethanol control induced only a slight reduction in mitochondrial activity, whereas 10% ethanol and 10% Tween 20 produced marked cell death. Therefore, the marked loss of viability observed for CUR-EtOH extract cannot be attributed solely to the solvent and reflects the biological effect of the tested extract under the present conditions. Nevertheless, because the residual ethanol content of the reconstituted CUR-EtOH preparation was not analytically quantified, a partial contribution from solvent carryover cannot be completely excluded. This pattern suggests a clear concentration- and time-dependent loss of cell metabolic activity in the presence of CUR-EtOH extract.

#### 3.4.2. Cytotoxicity Evaluation

**Figure 3:**
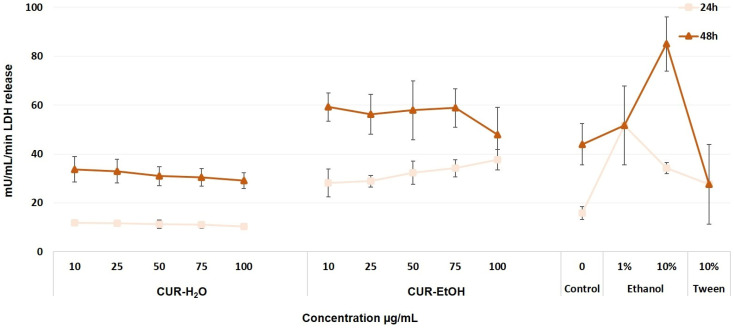
Lactate dehydrogenase release of cells treated with CUR-H_2_O and CUR-EtOH extracts. Values are expressed as mean ± SD. Statistical significance was evaluated by one-way ANOVA followed by Tukey’s post hoc test.

To complement the MTT results, the LDH release assay was used to evaluate membrane damage, since increased extracellular LDH is a marker of loss of membrane integrity and cell injury. Lactate dehydrogenase (LDH) release assay is a measure of the membrane integrity of cells, and indirectly correlates with cell death by necrosis. Thus, while the MTT assay reflects the metabolic competence of the cells, the LDH assay provides complementary information on whether the treatment causes structural membrane damage. In Figure 3, it can be observed that at 24 h, there is a baseline of LDH release that reaches 15.8 mU/mL/min, and this value is increased at 48 h to 44 mU/mL/min of LDH. Compared to these values at each time point, the status of the population viability can be inferred.

The CUR-H_2_O extract does not determine significant changes in LDH release at 24- and 48 h time-points, compared to the untreated controls of 24 and 48 h (*p* > 0.05). The absence of relevant LDH increases in the CUR-H_2_O-treated groups supports the MTT findings and suggests that this extract did not induce major membrane damage in HaCaT cells.

The CUR-EtOH extract determined a significant increase in LDH at both time points. At the 24 h time point (compared to the untreated control), this difference was noted in a concentration-dependent manner, increasing the LDH with the increased concentration (28.1–37.6 mU/mL/min). These increases were similar to the ones observed for the cell death controls (10% ethanol and 10% Tween 20: 34.2 and 27.5 mU/mL/min, no statistical intergroup difference, *p* > 0.05). At the 48 h point, LDH was high compared to the untreated control, but independent of concentration (59.2–56.2 mU/mL/min, *p* < 0.05). A slight reduction in this LDH release was noted at the highest concentration (47.9 mU/mL/min for the 100 µ/mL of CUR-EtOH extract), which could indicate a decline in population numbers. The higher LDH values recorded for CUR-EtOH extract confirm that the decrease in MTT signal was associated with real cell injury and not only with a transient metabolic slowdown.

#### 3.4.3. Wound Healing Assay

**Figure 4:**
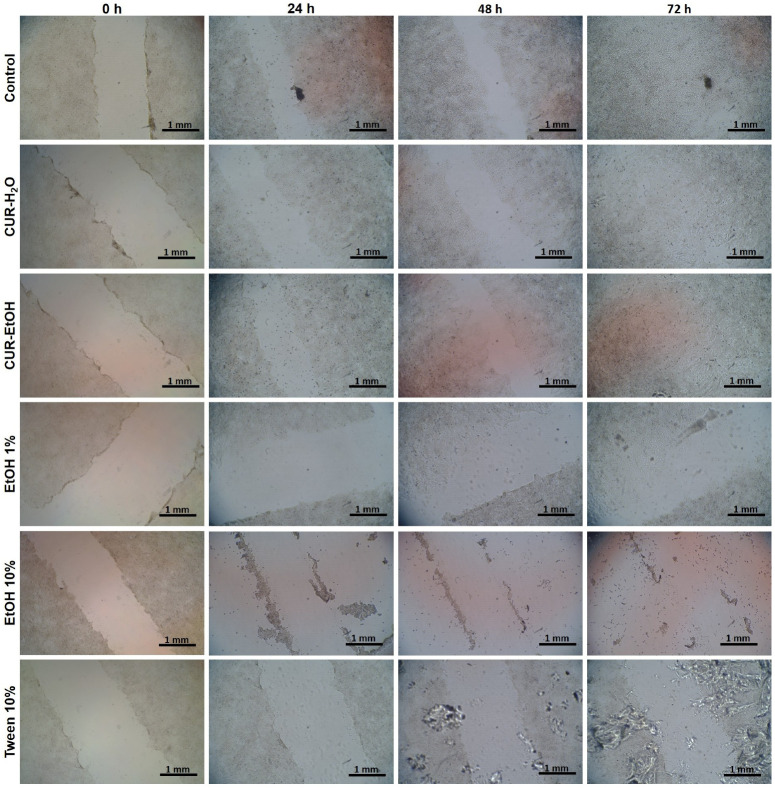
Representative wound-healing assay images for the untreated control, solvent controls, and the tested extracts (CUR-H_2_O and CUR-EtOH) at 10 µg/mL, recorded at 0, 24, 48, and 72 h. Scale bars represent 1 mm.

Based on the MTT and LDH results, the lowest tested concentration (10 µg/mL) was selected for the wound-healing assay to evaluate cell migration under conditions that would minimize overt cytotoxicity, especially for CUR-EtOH extract. Figure 4 depicts the representative images of the wound-healing assay for each sample group treated with the extracts at 10 µg/mL and the controls, at 0, 24, 48, and 72 h. Higher concentrations were not included in the wound-healing assay because, especially for CUR-EtOH, the preceding viability and cytotoxicity data indicated that they would likely confound migration-related interpretation through overt cytotoxic effects. These migration-related results should therefore be interpreted as preliminary comparative findings, given the limited number of independent wound-healing replicates.

**Figure 5:**
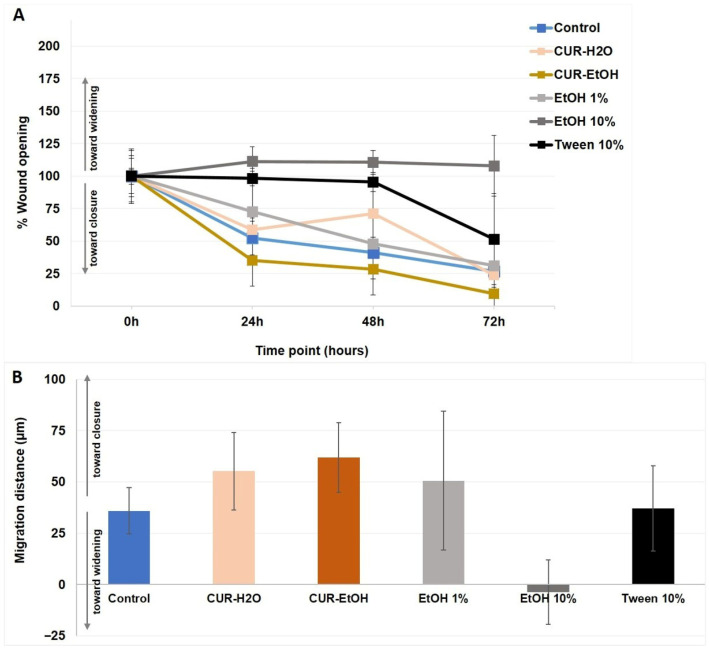
Wound healing assay graphs. (**A**) Percent of initial wound opening (0 h) for each tested extract and controls at 24, 48, and 72 h of exposure. (**B**) Migration distance covered by the cells that repopulated the free area at the end of the 72 h of exposure.

The wound-healing assay was used to assess the ability of HaCaT cells to migrate and repopulate a cell-free area after mechanical injury, thus providing a functional indicator of regenerative behavior in addition to simple viability measurements. The untreated control, as a baseline, showed a continuous repopulation of the initial gap, starting with the first 24 h, and ending with the almost complete closure of the gap at the end of the 72 h experiment (Figure 5A). The median distance migrated by growing cells was 35.8 ± 11.2 µm with a migration rate per day of 11.9 µm/day (Figure 5A,B). Wounds treated with the CUR-H_2_O extract at 10 µg/mL have almost closed by the last time point (72 h, Figure 4), covering a migration distance of 55.2 ± 18.7 µm at a pace of 18.4 µm/day (Figure 5A,B). Cells treated with CUR-EtOH extract showed the highest wound-closure rate to close the wound gap at the end of the 3 days (Figure 4): cells migrated a total distance of 61.9 ± 16.8 µm at a pace of 20.6 µm/day (Figure 5A,B). The EtOH 1% treated group presented similar good results: 50.5 ± 33.8 µm coverage at a pace of 16.8 µm/day. The 10% EtOH treated cells did not close the wound (Figure 4). On the contrary, the wound margins expanded as cells detached from the surface and ended up having a larger wound with −3.7 ± 15.6 µm, detaching at a pace of −1.2 µm/day (Figure 5A,B). Unexpectedly, the 10% Tween 20-treated group showed partial recovery of migration after 48 h (as the first two days had no relevant migration compared to the initial point), despite the high concentration of detergent (gap measuring 37.5 ± 20.6 µm, and an average of 12.3 µm/day).

Taken together, these data suggest that, at 10 µg/mL, both extracts supported wound repopulation, with CUR-EtOH showing the fastest migration-associated closure. However, this finding should not be interpreted as evidence of overall superior epithelial suitability, since CUR-EtOH also showed a markedly less favorable compatibility profile in the MTT and LDH assays at higher concentrations.

Overall, the in vitro data indicate that CUR-H_2_O extract was the more biocompatible extract toward HaCaT keratinocytes, whereas CUR-EtOH extract showed stronger cytotoxicity but also promoted faster wound-gap closure at the lowest tested concentration.

## 4. Discussion

The extraction yield obtained in the present study was 2.01% for CUR-H_2_O and 5.13% for CUR-EtOH, indicating a 2.55-fold higher gravimetric recovery for the ethanolic extract. This trend is consistent with the literature on *Curcuma longa*, where ethanolic or hydroethanolic systems generally provide higher recoveries than water, although absolute values vary according to the plant matrix, solvent composition, extraction conditions, and post-extraction processing [45,60,61,62,63,64]. Because the present study used commercially available turmeric powder rather than whole rhizome powder, these comparisons should be interpreted primarily as trend-based rather than numerically equivalent.

The lower performance of the aqueous extraction is also consistent with the poor water solubility of curcumin and the greater compatibility of this compound with organic solvents such as ethanol. In addition, the use of 96% ethanol likely favored the recovery of relatively hydrophobic constituents, whereas water was less efficient in recovering less polar turmeric-associated constituents. Overall, the lower crude yield observed here most likely reflects the combined effect of solvent selectivity, the turmeric-powder starting matrix, and the relatively mild maceration/ultrasound workflow [60,63,64,65,66,67,68]. At the same time, the post-maceration ultrasound step was not identical for the two extracts, since probe sonication was used for the ethanolic system and water-bath sonication for the aqueous system; therefore, the present comparison reflects the combined effect of solvent and applied extraction workflow rather than a perfectly parameter-matched solvent-only comparison. Although hydroalcoholic systems are often reported to enhance the extraction of phenolic and antioxidant-associated compounds, they were not included in the present study because the experimental design was intentionally restricted to two contrasting solvent conditions (water vs. 96% ethanol), as an initial solvent-dependent comparative model rather than a full extraction-optimization strategy.

The higher antioxidant activity of CUR-EtOH compared with CUR-H_2_O is consistent with the general trend reported for *Curcuma longa*, where ethanolic and especially hydroethanolic extracts usually outperform aqueous extracts in radical-scavenging assays. For example, Tanvir et al. reported that ethanolic turmeric extracts displayed stronger DPPH scavenging than the corresponding aqueous extracts, as reflected by markedly lower IC_50_ values (1.08–3.03 μg/mL for ethanolic extracts versus 5.31–16.55 μg/mL for aqueous extracts) [60]. Similarly, Toussé et al. observed that antioxidant activity increased from the aqueous to the ethanolic and hydroethanolic extract, with DPPH inhibition at 12.5 μg/mL of 7.91 ± 2.67%, 18.98 ± 2.81%, and 34.95 ± 2.05%, respectively, while the hydroethanolic extract reached 79.46 ± 2.12% inhibition at 200 μg/mL [69]. Taken together, these reports support the same solvent-dependent pattern observed in the present work. This comparison should be interpreted with caution, since the present DPPH assay was performed at a single concentration and therefore does not permit direct IC_50_-based comparison with studies using full concentration–response designs. The higher activity of the ethanolic extract is nevertheless chemically plausible. Curcumin is known to possess very poor aqueous solubility, around 0.6 μg/mL in pure water, whereas its solubility is much higher in organic solvents. This property strongly favors its extraction into ethanol and helps explain why ethanolic turmeric preparations generally show higher DPPH scavenging under the tested conditions than aqueous ones. These findings support the interpretation that ethanol promotes a better recovery of curcuminoids and other antioxidant constituents responsible for electron or hydrogen donation in the DPPH assay [70,71]. The fact that CUR-H_2_O extract still showed a relatively high DPPH inhibition despite the poor water solubility of curcumin may be explained by the prolonged 7-day maceration, the subsequent ultrasound treatment, and the use of lyophilized crude extract, which may contain finely dispersed turmeric-rich particles or other reducible constituents capable of reacting in the assay. Thus, while ethanol was clearly the more effective extraction medium in the present experiment, the aqueous protocol still produced an extract with appreciable antioxidant capacity.

The approximately 2.1-fold higher Folin–Ciocalteu response observed for CUR-EtOH compared with CUR-H_2_O is consistent with the general trend reported for *Curcuma longa*, where ethanolic or hydroethanolic solvents usually extract higher amounts of Folin–Ciocalteu-reactive compounds than water. In the study of Toussé et al., the aqueous extract contained 61.65 ± 1.58 mg GAE/g, the ethanolic extract 74.55 ± 2.37 mg GAE/g, and the hydroethanolic extract 133.19 ± 1.64 mg GAE/g, again showing a clear advantage of alcohol-containing extraction systems [69]. Likewise, Tanvir et al. reported total polyphenol values ranging from 4.52 to 7.68 g GAE/100 g in aqueous turmeric extracts and 6.15 to 16.07 g GAE/100 g in ethanolic extracts, which corresponds to approximately 45.2–76.8 mg GAE/g and 61.5–160.7 mg GAE/g, respectively [60]. These values should be interpreted cautiously, since the Folin–Ciocalteu assay reflects overall reducing capacity rather than exclusively true phenolic content, and curcuminoids themselves can substantially contribute to the measured response [72,73]. Accordingly, the higher TPC observed for CUR-EtOH should not be interpreted as direct proof of selective enrichment only in phenolic compounds, but rather as evidence of a stronger pool of Folin–Ciocalteu-reactive reducing constituents under the applied extraction conditions.

The higher TPC of CUR-EtOH extract can be explained primarily by solvent polarity and solute compatibility. Curcuminoids are relatively hydrophobic compounds, and ethanol is much more effective than water in solubilizing them. Choi et al. likewise showed that increasing the ethanol concentration increased the Folin–Ciocalteu response of turmeric extracts, with water (0% ethanol) producing only 1.19 ± 0.11 mg GAE/g dried turmeric, whereas ethanol-containing systems yielded progressively higher values [67]. In practical terms, this means that the higher TPC observed for CUR-EtOH extract in the present study is most likely due to better extraction of curcumin and structurally related antioxidant molecules into ethanol. This interpretation is also supported by the fact that the ethanolic extract showed higher DPPH scavenging in parallel with higher TPC, in line with the positive association between Folin–Ciocalteu values and antioxidant activity reported for turmeric extracts [67,69,73]. Therefore, the higher TPC observed for CUR-EtOH extract may reflect not only a greater recovery of phenolic constituents, but also solvent-dependent co-extraction of other Folin–Ciocalteu-reactive reducing compounds; accordingly, this result should be interpreted as evidence of a chemically richer reducing fraction rather than as absolute proof of selective phenolic enrichment alone.

The UHPLC-MS findings indicate that ethanol was more efficient than water in recovering the quantified low-molecular-weight phenolic acids from the turmeric-based matrix, particularly hydroxycinnamic acid derivatives. This pattern agrees with the broader phytochemical literature on *Curcuma longa*, although whole-rhizome extracts usually display a broader fingerprint than the one observed here [74,75]. The narrower profile detected in the present study is most likely explained by the use of commercially available turmeric powder rather than whole rhizome material, together with the targeted nature of the UHPLC-MS method, which was restricted to a predefined panel of compounds. The present results should be interpreted as evidence that ethanol was more efficient than water in recovering the quantified low-molecular-weight phenolic acids under the applied experimental conditions, rather than as a complete phytochemical characterization of turmeric-derived material. For these reasons, the lower number of detected compounds and the lower absolute concentrations observed here should be interpreted primarily as a reflection of the different starting matrix, different extraction system, and different analytical strategy [73,74,76]. Accordingly, the high number of NF and <LOQ entries narrows the scope of the chromatographic comparison, but does not invalidate it, since the solvent-dependent interpretation was based only on the subset of analytes that were reliably detectable under the applied analytical conditions. The higher concentrations observed in the CUR-EtOH extract are also chemically plausible and are in line with solvent-extraction studies on turmeric. Ethanol-containing systems generally recover phenolic antioxidants from *Curcuma longa* more efficiently than water, and ethanol concentration has been identified as one of the most influential variables affecting extraction performance [67]. In the present study, the use of 96% ethanol likely favored the extraction of relatively less polar phenolic acids and turmeric-associated reducing compounds, whereas the aqueous extract was limited by the lower compatibility of these compounds with water. This solvent effect explains why p-coumaric derivatives were about 6.5-fold higher and ferulic derivatives about 9.2-fold higher in CUR-EtOH than in CUR-H_2_O, and why dihydroxybenzoic acid derivatives were only detectable in the ethanolic extract. Because curcuminoids were not included in the validated analytical panel used in this study, the UHPLC-MS data presented here cannot be interpreted as evidence of their absence from the extracts, but only as a targeted assessment of selected non-curcuminoid polyphenols.

The UHPLC findings also fit well with the higher total phenolic content and DPPH scavenging activity observed for CUR-EtOH in the present study. Phenolic acids such as p-coumaric and ferulic acid belong to the hydroxycinnamic acid class, which is widely recognized for its reducing and radical-scavenging properties. Their enrichment in the ethanolic extract likely contributed, at least in part, to the superior antioxidant profile of CUR-EtOH. At the same time, the fact that several target compounds were not found or were detected below the quantification threshold suggests that the antioxidant activity of the extracts was not driven exclusively by the few quantified phenolic acids, but also by other turmeric-related or reducing constituents that were either present below the analytical threshold or not included in the targeted UHPLC panel. Thus, the chromatographic data support the view that ethanol provided a chemically richer antioxidant fraction than water, while also highlighting that the present extracts differ from conventional turmeric rhizome extracts because they originated from commercially available turmeric powder rather than directly from freshly processed whole rhizome material [74,76,77].

In the present study, both turmeric-powder-derived preparations exhibited antimicrobial activity, but their efficacy was clearly strain- and solvent-dependent. The ethanolic extract (CUR-EtOH) showed the strongest antibacterial effect against the oral streptococci, with a MIC of 10 µL for *Streptococcus mutans* and 100 µL for *Streptococcus oralis*, whereas the aqueous extract (CUR-H_2_O) showed a MIC of 60 µL against *S. mutans* and no detectable MIC against *S. oralis* within the tested range. By contrast, against *Candida albicans*, both extracts were active, but the aqueous extract was slightly more effective, with a MIC of 60 µL compared with 80 µL for the ethanolic extract. This pattern suggests that CUR-EtOH extract was more effective against the tested Gram-positive oral bacteria, while CUR-H_2_O extract retained a moderate antifungal effect under the present assay conditions. Because the present antimicrobial assay was restricted to planktonic broth microdilution screening, the results should be interpreted as preliminary comparative evidence and not as direct proof of anti-biofilm efficacy in oral conditions.

The higher antibacterial activity of CUR-EtOH extract against *S. mutans* is consistent with the literature on curcumin and turmeric-derived preparations. Curcumin has previously been shown to inhibit *S. mutans* growth at a MIC of 128 µg/mL and, importantly, to reduce bacterial adherence to extracellular matrix proteins and human tooth surfaces even at sub-MIC levels. In addition, curcumin has been reported to reduce *S. mutans* biofilm formation by inhibiting sortase A activity, a key virulence-associated enzyme involved in bacterial adhesion and biofilm development. Therefore, the stronger effect of CUR-EtOH extract observed here is biologically plausible and supports the view that ethanol is more efficient than water in recovering antibacterial constituents with anti-cariogenic potential from a turmeric-based matrix [78,79,80]. With respect to *S. oralis*, the present findings indicate that this strain was less susceptible than *S. mutans*, especially to the aqueous extract, for which no MIC could be established in the tested range. Comparable data for *S. oralis* are much scarcer than for *S. mutans*, but a recent in vitro study using a hydroalcoholic turmeric extract reported dose-dependent inhibition zones of 22.0 ± 0.5, 16.0 ± 3.5, 12.0 ± 1.5, and 10.0 ± 1.75 mm against *S. oralis* at 1000, 500, 200, and 100 µg/mL, respectively, while ampicillin showed a larger inhibition zone (30.0 ± 4.0 mm). The same paper explicitly noted that published data on turmeric against *S. oralis* remain sparse. This pattern in the literature agrees well with the present results, where *S. oralis* responded to CUR-EtOH extract only at the highest tested volume and remained poorly sensitive to CUR-H_2_O extract [81].

The antifungal findings also fit with previous reports showing that *Curcuma longa* and curcumin possess anti-Candida activity, although the quantitative outcomes vary substantially across studies. Abdelgader et al. reported that an alcoholic extract of turmeric completely inhibited *C. albicans* growth at 800 µL, with a minimum fungicidal concentration of 1600 µL [82]. Other studies have reported that turmeric methanolic extracts can inhibit *C. albicans* with MIC values around 256 µg/mL, while curcumin itself has shown very broad MIC ranges depending on the strain tested, from 250 to 500 µg/mL in some standard strains to 1000 to >5000 µg/mL in clinical isolates; conversely, much lower MICs (7.8–32.25 µg/mL) have been reported for selected nystatin-resistant strains [83,84,85]. This wide variation indicates that antifungal susceptibility is strongly influenced by the fungal strain, the formulation tested, and the assay design. In this context, the moderate activity of both CUR-EtOH and CUR-H_2_O extracts observed in the present study is well within the broad range of antifungal responses reported for turmeric-based systems.

Although alcohol-based turmeric extracts often show stronger antimicrobial effects than aqueous preparations, the slightly better activity of CUR-H_2_O extract against *C. albicans* observed here is still biologically plausible. First, antifungal activity does not necessarily correlate directly with total phenolic content or DPPH activity, because different compounds may act through distinct microbial targets. Second, the two extracts likely differed not only in total yield and polyphenol profile but also in the relative abundance of hydrophilic versus less polar constituents, which may affect fungal cells differently from bacterial cells. Third, our antimicrobial results were expressed as µL of stock extract added per well, whereas many published studies report µg/mL, mg/mL, or inhibition-zone diameters; therefore, direct numerical comparison is limited, and the most reliable interpretation is based on comparative trends rather than one-to-one equivalence with published MIC values. The superior antibacterial effect of CUR-EtOH extract against *S. mutans* and *S. oralis* is likely related to solvent selectivity and extraction chemistry. Curcumin and several associated turmeric constituents are more efficiently recovered in ethanol-containing systems than in water, resulting in extracts richer in lipophilic antibacterial molecules. This interpretation is also coherent with our own chemical data, where CUR-EtOH extract showed higher TPC and a stronger antioxidant response than CUR-H_2_O extract. Nevertheless, antimicrobial activity cannot be attributed exclusively to total phenolics, because curcumin itself, ferulic acid, p-coumaric acid, and other co-extracted compounds may contribute synergistically to membrane disruption, enzyme inhibition, and antibiofilm effects. Studies on curcumin antimicrobial action further indicate that this molecule tends to act more strongly against Gram-positive bacteria than against many other microorganisms, whereas *Candida* susceptibility is more variable and formulation-dependent [80,83,84].

As noted above, the present findings must also be interpreted in light of the starting matrix, since the extracts were prepared from commercially available turmeric powder rather than whole rhizome powder. In addition, our broth microdilution assay with extract-background correction differs methodologically from agar diffusion and biofilm-based studies, which further limits direct numerical comparison with the literature.

The in vitro biological assessment highlighted a clear divergence between the two turmeric-powder-derived extracts in terms of keratinocyte compatibility. Although HaCaT cells provide a practical epithelial model for comparative cytocompatibility and migration screening, they are skin-derived keratinocytes and therefore do not fully reproduce the specific biology of oral mucosa. Overall, CUR-H_2_O extract showed the more favorable biocompatibility profile, maintaining HaCaT mitochondrial activity close to or above the untreated control at both 24 and 48 h, without inducing relevant LDH release, whereas CUR-EtOH extract caused a marked concentration- and time-dependent reduction in mitochondrial activity together with increased LDH release, indicating true cell injury rather than a transient metabolic slowdown. At the same time, when the concentration was reduced to 10 µg/mL, both extracts still allowed wound repopulation in the scratch assay, with CUR-EtOH extract showing the fastest migration rate and CUR-H_2_O extract also improving closure compared with the untreated control. Thus, the present data suggest a dose-dependent dual behavior, in which the aqueous extract showed a more favorable compatibility profile in HaCaT keratinocytes, while the ethanolic extract retained a biologically active fraction that could still stimulate migration under low-exposure conditions. However, this interpretation should be considered within the limits of the experimental model, since HaCaT cells represent an immortalized skin keratinocyte line rather than oral mucosal epithelial cells. Therefore, the present findings support a preliminary epithelial compatibility and migration profile, but direct extrapolation to the oral mucosa should be made with caution. This pattern is broadly consistent with the published literature on curcumin and keratinocytes, which indicates that curcumin may behave in a biphasic manner depending on dose, formulation, and cell context. Zhao et al. reported that low concentrations of curcumin (2.5 and 5 μM) protected HaCaT keratinocytes against arsenite-induced acute cytotoxicity and improved cell survival through an NRF2-dependent mechanism, supporting the idea that curcumin can exert cytoprotective and antioxidant effects under controlled exposure conditions [86]. Likewise, Chen et al. showed that curcumin improved keratinocyte proliferation, inflammation, and oxidative stress in a UV-induced actinic dermatitis model, again suggesting that curcumin can be beneficial to keratinocyte biology in a stress-related setting [87]. In contrast, other studies have shown that higher concentrations are distinctly anti-proliferative or pro-apoptotic. Lundvig et al. reported that high doses of curcumin triggered cytotoxic responses in HaCaT keratinocytes, while Song et al. demonstrated that curcumin markedly inhibited HaCaT growth, arrested cells in the G2/M phase, and induced apoptosis, with an apoptosis rate of 33.95% after 24 μM curcumin treatment [88,89]. Taken together, these studies support the interpretation that the biological profile observed here is not contradictory, but rather reflects the well-known concentration-dependent switch of curcumin from a protective/redox-active compound to a cytotoxic one. From this perspective, the better tolerance of CUR-H_2_O extract and the stronger cytotoxicity of CUR-EtOH extract are also mechanistically plausible in light of your own chemical data. The ethanolic extract had the highest extraction yield, the highest antioxidant potential, the highest total phenolic content, and a clear enrichment in p-coumaric and ferulic acid derivatives compared with the aqueous one. At the same time, the solvent control experiment showed that 1% ethanol reduced mitochondrial activity only modestly (to ~89%), whereas the ethanolic extract reduced viability far more strongly, indicating that the biological effect cannot be attributed to residual ethanol alone. Therefore, the 96% ethanol recovered a more concentrated fraction of hydrophobic, turmeric-rich, and phenolic-active constituents, which likely enhanced both the beneficial redox-related properties and the cytotoxic pressure on normal keratinocytes. This fits well with the fact that the material used for the extraction process was turmeric powder rather than whole rhizome, meaning that the ethanolic extract was probably particularly enriched in curcuminoid-type molecules rather than in a broader phytochemical matrix that might otherwise dilute or buffer their effects. The LDH data strongly reinforce this interpretation. While the MTT assay alone may sometimes reflect reduced metabolic activity without irreversible death, the simultaneous increase in extracellular LDH after CUR-EtOH extract exposure indicates that the ethanolic extract compromised membrane integrity and produced real cytotoxicity in HaCaT cells. This is again in line with the literature showing that unformulated curcumin can be harmful to keratinocytes at sufficiently high intracellular exposure, whereas improved delivery systems may preserve beneficial effects while reducing toxicity. For example, Nair et al. reported that curcumin-encapsulated chitosan nanoparticles showed higher HaCaT viability than curcumin solution, highlighting the importance of formulation in modulating keratinocyte tolerance [90]. Therefore, one important implication is that the biological potential of the ethanolic extract may not be fully exploitable in its current crude form for direct epithelial exposure, but could become more attractive if reformulated into a controlled-release or topical delivery system.

Despite its poorer overall biocompatibility, CUR-EtOH extract still produced the fastest wound-gap closure at 10 µg/mL, whereas CUR-H_2_O extract also improved migration relative to the untreated control. This suggests that once the concentration is kept within a lower, non-overwhelming range, both extracts may support at least some aspects of keratinocyte motility and repopulation. Ang et al. reported that native and microencapsulated curcuminoids showed limited effects on HaCaT migration and proliferation in one in vitro model, indicating that curcuminoids do not necessarily stimulate scratch closure in every experimental setting [91]. In contrast, Zhou et al. showed that Pluronic F127-liposome-encapsulated curcumin promoted HaCaT migration through activation of the Nrf2/Keap1 pathway, and Sharma et al. reported wound-healing efficacy for curcumin conjugated to hyaluronic acid in vitro and in vivo [92,93]. Taken together, these studies suggest that curcumin-related wound-healing behavior is strongly dependent on dose, delivery system, and cellular context. A crude ethanolic extract may be cytotoxic at moderate-to-high concentrations yet still stimulate cell migration at a sufficiently low concentration, while the aqueous extract shows a safer but somewhat less stimulatory profile. Accordingly, the biological effects observed in the present study may reflect not only curcuminoid-related activity, but also the contribution of non-curcuminoid molecules and broader solvent-dependent matrix interactions, which could not be fully dissected by the applied analytical design.

For oral-health-oriented applications, the ideal preparation should combine host–cell compatibility with activity against oral pathogens. In the present work, the CUR-EtOH extract was clearly more active against *S. mutans* and *S. oralis*, whereas the CUR-H_2_O extract was more biocompatible toward HaCaT cells and slightly more active against *C. albicans*. Therefore, an interesting functional distinction is created between the two extracts. On the one hand, the ethanolic extract may be the more promising option when the priority is a stronger antibacterial effect against cariogenic bacteria; on the other hand, the aqueous extract may be the more suitable candidate when epithelial safety and tolerance are more important, particularly for repeated topical or mucosal exposure. More broadly, the coexistence of antioxidant activity and wound-gap closure in the present study should not be interpreted as direct mechanistic proof that the former caused the latter. Rather, these findings represent parallel functional observations that may be biologically related, but this relationship was not mechanistically demonstrated in the present experimental design. Importantly, the apparently superior wound-gap closure induced by CUR-EtOH extract at 10 µg/mL should not be interpreted as evidence of overall superior epithelial suitability, because this extract showed clear cytotoxicity at higher concentrations in both the MTT and LDH assays. Therefore, the migration-promoting effect of CUR-EtOH extract appears to be condition-dependent and restricted to low-exposure settings, rather than indicative of a generally better biological profile. Moreover, the faster wound-gap closure observed under these conditions may reflect not only cell migration, but also a possible contribution from proliferation-related or adaptive compensatory responses, which were not mechanistically dissected in the present study. Our results suggest that the CUR-H_2_O extract may offer a better safety-oriented profile, whereas the CUR-EtOH extract may represent the more potent but narrower therapeutic-window extract, whose practical use would likely benefit from dose optimization or formulation improvement [80,94,95]. At this stage, however, the tested in vitro exposure levels should be regarded as comparative screening conditions rather than direct predictors of clinically achievable intraoral doses. Accordingly, the novelty of the present work lies less in the botanical source itself and more in the integrated solvent-dependent comparison of turmeric-powder-derived preparations within an oral-health-oriented experimental framework.

## 5. Limitations of the Study

The present study has several limitations that should be considered when interpreting the findings. First, the extracts were prepared from commercially available turmeric powder derived from *Curcuma longa* rather than from whole rhizome powder, which limits the direct comparability of the extraction yield, phytochemical profile, and biological activity data with many published studies performed on crude plant material. Second, the post-maceration ultrasound-assisted processing was not fully identical for the two extracts, since the ethanolic sample was treated by probe sonication and the aqueous sample by ultrasonic water bath, with different treatment durations. Therefore, the observed differences should be interpreted as reflecting the combined influence of solvent choice and solvent-specific extraction workflow, rather than the isolated effect of the solvent alone. Third, no hydroalcoholic extraction system was included. Since hydroethanolic mixtures often provide improved recovery of phenolic constituents, their inclusion would have strengthened the solvent-comparison framework. Therefore, the present study should be interpreted as a comparison between two contrasting solvent conditions rather than as a complete solvent-optimization analysis. Fourth, the chemical characterization was based on DPPH, Folin–Ciocalteu, and a targeted UHPLC-MS panel, which, although informative, does not provide a complete characterization of the extract composition. In addition, extraction efficiency was not monitored over time, and the 7-day maceration period was adopted from the applied extraction protocol rather than established through a dedicated optimization study. The DPPH assay was performed at a single working concentration, and no concentration–response curve or IC_50_ value was established. Therefore, the antioxidant comparison should be interpreted as a relative screening endpoint rather than as a full potency assessment. The Folin–Ciocalteu assay reflects overall reducing capacity rather than exclusively true phenolic content, while the UHPLC-MS analysis was restricted to a predefined set of compounds and did not include a full quantification of the major curcuminoids. In addition, the Folin–Ciocalteu assay lacks full specificity for phenolic compounds and may also reflect interference from other reducing constituents; therefore, the reported TPC values should be interpreted as operational reducing-capacity estimates rather than as absolute phenolic quantification. Moreover, the higher TPC measured in the ethanolic extract may partly reflect solvent-dependent co-extraction of nonphenolic reducing constituents, since the Folin–Ciocalteu assay does not provide absolute specificity for phenolic compounds. The UHPLC-MS characterization was restricted to a targeted 15-compound polyphenolic panel and did not include curcuminoids; therefore, the chromatographic data should be interpreted as partial phytochemical information rather than a complete compositional characterization. Moreover, the large number of NF and <LOQ results further indicates that the UHPLC-MS data provide only partial targeted phytochemical information. Consequently, the comparative chromatographic conclusions should be interpreted as limited to the analytes detectable within the applied panel and method sensitivity.

A further limitation concerns the antimicrobial testing design. The antimicrobial activity was assessed against only three reference strains and under planktonic growth conditions, which does not fully reproduce the complexity of oral microbial communities or biofilm-related behavior. In addition, MIC values were expressed as the lowest tested volume of stock extract per well, rather than as standardized final concentrations in mg/mL or µg/mL, which limits direct numerical comparison with the wider literature. The antimicrobial assays also did not investigate bactericidal/fungicidal endpoints, antibiofilm activity, or possible synergistic effects with conventional antimicrobial agents. Although ampicillin appeared in the raw laboratory worksheet as a bacterial reference condition, no matched set of standard antibacterial and antifungal comparator agents was included across the full microbial panel; therefore, the antimicrobial results were interpreted primarily as a comparative extract-versus-extract assessment. The antimicrobial evaluation was performed only on planktonic cultures and did not include biofilm inhibition or biofilm-disruption assays. Given the central role of biofilms in oral infections, future studies should investigate these extracts in single-species, multispecies, and interkingdom biofilm models.

The in vitro biological assessment also has important limitations. The cytocompatibility and migration experiments were performed on HaCaT cells, which represent an immortalized skin keratinocyte model rather than oral mucosal epithelial cells; therefore, extrapolation to the oral environment should be made with caution. The present in vitro findings should be interpreted as preliminary epithelial screening data that require validation in oral keratinocyte-based models. In addition, the wound-healing assay was performed at a single concentration (10 µg/mL) selected on the basis of MTT/LDH pre-screening; therefore, no dose–response relationship for migration/repopulation could be established in the present study. Although multiple measurements were taken within each image, these do not substitute for independent biological replicates; therefore, the migration-related statistical inferences should be interpreted cautiously and confirmed in future studies with larger sample sizes. The biological assays were further limited to short-term endpoints (24–48 h for MTT and LDH; up to 72 h for wound healing), without mechanistic investigations into oxidative stress modulation, apoptosis, inflammation-related pathways, or epithelial barrier-related markers. Therefore, the wound-healing results cannot distinguish whether enhanced gap closure was driven predominantly by migration, proliferation, or compensatory stress-related responses. In addition, although ethanol controls were included in the cell-based assays, the residual ethanol content of the reconstituted CUR-EtOH extract was not analytically quantified. Therefore, a partial solvent-related contribution to the observed cytotoxicity cannot be completely excluded.

Another limitation is that no specific mechanistic markers of apoptosis, necrosis, or intracellular oxidative stress were evaluated. Therefore, the cytotoxicity observed in the MTT and LDH assays should be interpreted as functional evidence of reduced viability and membrane injury, rather than as definitive proof of a specific cell-death pathway.

Finally, the present work should be regarded as a preliminary preclinical study, since no formulation optimization, stability testing, permeability assessment, or in vivo validation was performed. Therefore, although the findings support the potential relevance of these turmeric-powder-derived extracts for future oral-health-oriented applications, additional studies are required to confirm their efficacy, safety, and translational value under more clinically relevant conditions.

## 6. Conclusions

The present study demonstrated that the extraction solvent strongly influenced both the chemical profile and the biological behavior of the turmeric-powder-derived extracts. CUR-EtOH extract showed higher extraction yield, antioxidant activity, total phenolic content, and a richer targeted polyphenolic profile than CUR-H_2_O extract. From a microbiological perspective, CUR-EtOH extract was more effective against the tested oral streptococci, especially *S. mutans*, whereas CUR-H_2_O extract showed a slightly better antifungal effect against *C. albicans*. In the HaCaT model, CUR-H_2_O extract exhibited the more favorable compatibility profile, whereas CUR-EtOH extract showed stronger cytotoxicity, despite promoting faster wound-gap closure at 10 µg/mL under low-exposure conditions. Accordingly, the wound-healing-related effect of CUR-EtOH extract should be interpreted within the limits of its narrower in vitro safety profile. Overall, the findings suggest that the two extracts may be better suited to different applications: CUR-EtOH extract appears more promising for antimicrobial-oriented strategies, whereas CUR-H_2_O extract may represent the safer option when epithelial compatibility is prioritized. These findings should be interpreted as preliminary comparative evidence that may support the future rational design of turmeric-based oral formulations, rather than as direct proof of immediate formulation superiority.

## Figures and Tables

**Table 1:** Concentration of the targeted polyphenolic compounds included in the UHPLC-MS panel present in the CUR-H_2_O and CUR-EtOH extracts.

Name	Concentration of the CUR-EtOH Extract [µg/mL]	Concentration of the CUR-H_2_O Extract [µg/mL]	*m*/*z*	Retention Time
p-Coumaric acid/trans-p-Coumaric acid	5.24 ± 0.03 *	0.81 ± 0.07	163.0500 > 119.0500	4.516
(E/Z)-Ferulic acid/trans-Ferulic acid	5.15 ± 0.02 *	0.56 ± 0.01	193.0600 > 134.1000	4.916
3,5-Dihydroxybenzoic Acid/3,4-Dihydroxybenzoic Acid	0.46 ± 0.09	NF	153.0300 > 108.9000	1.157
Rutin	<LOQ	<LOQ	609.1500 > 300.1000	5.3
Quercetin	<LOQ	<LOQ	301.0400 > 151.1000	5.90
2,4-Dihydroxybenzoic Acid	NF	NF	153.0300 > 108.9000	2.376
Caffeic acid	NF	NF	179.0400 > 135.0500	2.999
Chlorogenic acid	NF	NF	353.1000 > 191.2000	2.574
Rosmarinic acid	NF	NF	359.0800 > 161.1000	5.5
Gallic acid	NF	NF	169.1000 > 125.0500	0.699
Resveratrol	NF	NF	227.0000 > 143.0000	5.369
Genistein	NF	NF	269.0000 > 133.0000	6.106

Note: NF—not found; below the limit of detection; LOQ—limit of quantification. Values are expressed as the mean ± SD (*n* = 3). * *p* < 0.05 compared with CUR-H_2_O for the same compound.

**Table 2:** Mean optical density (OD) values of microbial cultures exposed to CUR-EtOH and CUR-H_2_O extracts at different tested volumes.

Conc. [μL]	*S. mutans* ATCC 25175				*S. oralis* ATCC 9811				*C. albicans* ATCC 10231			
	CUR-EtOH		CUR-H_2_O		CUR-EtOH		CUR-H_2_O		CUR-EtOH		CUR-H_2_O	
	OD	SD	OD	SD	OD	SD	OD	SD	OD	SD	OD	SD
10	1.961	0.007	3.002	0.002	2.658	0.001	2.992	0.008	2.309	0.003	2.776	0.006
20	2.458	0.009	3.207	0.060	3.012	0.030	3.126	0.001	2.73	0.004	3.089	0.000
40	2.777	0.008	3.727	0.007	3.15	0.003	3.513	0.002	2.971	0.007	3.48	0.007
60	2.998	0.006	3.569	0.003	3.376	0.006	3.635	0.004	3.203	0.001	3.525	0.004
80	3.144	0.016	3.78	0.008	3.479	0.025	3.863	0.003	3.305	0.035	3.759	0.002
100	3.464	0.002	3.876	0.001	3.54	0.010	3.950	0.004	3.565	0.016	3.859	0.005

**Table 3:** Corrected optical density values (OD_sample_ − OD_extract_ background) for microbial cultures treated with CUR-EtOH and CUR-H_2_O extracts.

Conc. [μL]	*S. mutans* ATCC 25175		*S. oralis* ATCC 9811		*C. albicans* ATCC 10231	
	CUR-EtOH	CUR-H_2_O	CUR-EtOH	CUR-H_2_O	CUR-EtOH	CUR-H_2_O
10	0.351	0.830	1.048	0.820	0.699	0.604
20	0.3105	0.686	0.864	0.605	0.582	0.567
40	0.2985	0.646	0.671	0.433	0.492	0.399
60	0.244	0.357	0.622	0.423	0.449	0.313
80	0.205	0.325	0.54	0.408	0.365	0.304
100	0.2095	0.293	0.285	0.366	0.31	0.276

**Table 4:** Growth/inhibition percentages relative to the positive growth control and minimum inhibitory concentration (MIC) of CUR-EtOH and CUR-H_2_O extracts against the tested microbial strains.

Conc. [μL]	*S. mutans* ATCC 25175				*S. oralis* ATCC 9811				*C. albicans* ATCC 10231			
	CUR-EtOH		CUR-H_2_O		CUR-EtOH		CUR-H_2_O		CUR-EtOH		CUR-H_2_O	
	BGR [%]	BIR [%]	BGR [%]	BIR [%]	BGR [%]	BIR [%]	BGR [%]	BIR [%]	MGR [%]	MIR [%]	MGR [%]	MIR [%]
10	94.1	5.9	222.7	0.0	306.6	0.0	239.8	0.0	183.5	0.0	158.7	0.0
20	83.2	16.8	183.9	0.0	252.8	0.0	176.9	0.0	152.8	0.0	149.0	0.0
40	80.0	20.0	173.3	0.0	196.2	0.0	126.6	0.0	129.1	0.0	104.9	0.0
60	65.4	34.6	95.8	4.2	182.0	0.0	123.8	0.0	118.0	0.0	82.3	17.7
80	55.0	45.0	87.1	12.9	157.9	0.0	119.3	0.0	95.9	4.1	79.9	20.1
100	56.2	43.8	78.6	21.4	83.3	16.7	107.2	0.0	81.4	18.6	72.4	27.6
MIC [μL]/MIC [mg/well]												
*S. mutans* ATCC 25175					*S. oralis* ATCC 9811				*C. albicans* ATCC 10231			
CUR-EtOH			CUR-H_2_O		CUR-EtOH		CUR-H_2_O		CUR-EtOH		CUR-H_2_O	
10/0.978			60/5.892		100/9.780		ND/ND		80/7.824		60/5.892	

When the corrected OD of the tested sample exceeded that of the positive growth control, inhibition was recorded as 0.0%, indicating the absence of inhibitory activity under the applied experimental conditions. Growth values >100% reflect that the corrected OD of the tested sample was higher than that of the positive growth control and should not be interpreted as true stimulation of microbial growth. MIC values are expressed as the lowest tested stock-extract volume per well associated with growth inhibition. Based on stock concentrations of 97.8 mg/mL (CUR-EtOH) and 98.2 mg/mL (CUR-H_2_O), the tested volumes of 10, 20, 40, 60, 80, and 100 µL correspond to 0.978, 1.956, 3.912, 5.868, 7.824, and 9.780 mg/well for CUR-EtOH and 0.982, 1.964, 3.928, 5.892, 7.856, and 9.820 mg/well for CUR-H_2_O, respectively.

## Data Availability

All the data obtained are included in the present article. Further inquiries can be directed to the corresponding author.

## Abbreviations

The following abbreviations are used in this manuscript:

CUR-H_2_O	Aqueous extract prepared from commercially available turmeric powder (*Curcuma longa*)
CUR-EtOH	Ethanolic extract prepared from commercially available turmeric powder (*Curcuma longa*)
DPPH	1,1-diphenyl-2-picrylhydrazyl
TPC	Total Phenolic Content
AOP	Antioxidant Potential
UHPLC	Ultra-high-performance liquid chromatography
GAE	Gallic acid equivalents
ESI	Electrospray ionization source
MS	Mass spectrometry
MIC	Minimum inhibitory concentration
OD	Optical density
ATCC	American Type Culture Collection
SDA	Sabouraud Dextrose Agar
BHI	Brain Heart Infusion
BGR/MGR	Bacterial/mycelial growth rate
BIR/MIR	Bacterial/mycelial inhibition rate
HaCaT	Immortalized human keratinocyte cell line
MTT reagent	3-[4,5-dimethylthiazol-2-yl]-2,5-diphenyl tetrazolium bromide
LDH	Lactate dehydrogenase
LOQ	Limit of quantification
ND	Not detected
NF	Not found
